# The Influence of Parental Educational Expectations on Children’s Higher Education Attainment: Re-estimation Based on Instrumental Variables

**DOI:** 10.3389/fpsyg.2022.899348

**Published:** 2022-05-17

**Authors:** Ting Lai, Fulan Liu, Yiheng Huang

**Affiliations:** ^1^School of Economics, Shenzhen University, Shenzhen, China; ^2^Foreign Languages College, Jiangxi Normal University, Nanchang, China; ^3^China Center for Special Economic Zone Research, Shenzhen University, Shenzhen, China

**Keywords:** parental educational expectations, higher education attainment, family background, college enrolment opportunity index, instrumental variables

## Abstract

Studies show that parental educational expectations (PEEs) serve as an intermediary variable between family background and children’s educational attainment. This paper re-examines the relationship between PEEs and children’s higher educational attainment using data from the China Family Panel Studies (CFPS) 2010–2018. To address potential endogenous problems in the previous papers, we use the average College Enrolment Opportunity Index (CEOI) when the children were 10–12 years old as an instrumental variable for PEEs. The results revealed that: (1) In addition to the indirect intermediary effects, the PEEs also had a direct impact on children’s higher educational attainment independent of family background; (2) the magnitude of the effect was much larger (almost three times) than previous estimates after solving endogenous problems; (3) there was no significant gender difference in the effect of PEEs. In addition, we also found that PEEs had a greater impact on middle- and low-income families. Therefore, we argue that against the background of the “Double Reduction” policy, parents should change their conception of education and raise their expectations for their children and encourage them to strive for higher educational achievements.

## Introduction

In the classic model of human capital by Becker (1994), education is an investment, and the profit that an individual makes from receiving an education is the difference between the cash flow of future earnings and the cost of education (time, money, etc.) (Becker, 1994). Education is both an important factor in increasing labor productivity and an important channel for social mobility. Thus, the significance of exploring the factors that influence educational attainment is twofold (Cham et al., 2014). As a core component of human capital, education is an important causative factor for gaining social status and a key mechanism for alleviating social inequality. However, education is also a reproduction mechanism for the transmission of class dominance (Blau and Duncan, 1967).

In the discussion of factors that affect educational attainment, the Wisconsin School, based on the famous Blau-Duncan Status Attainment Model, argues that as an important mediating variable, parental educational expectations (PEEs) have a significant influence between family background and children’s educational attainment. Many scholars have explored the influence of PEEs on children’s educational attainment from the perspective of intermediate effects (e.g., Liu et al., 2015; Wang et al., 2018). However, some scholars have argued that PEEs also have an independent impact on children’s educational attainment. For example, Lu et al. (2021) revealed the direct effect of PEEs on adolescents’ academic performance and claimed that these effects are greater for migrant, one-child and non-poor families. Similarly, by investigating China’s left-behind children, Zhang et al. (2020) revealed the direct effect of PEEs on their children’s learning input. Most of the papers focused on particular groups, but it remains unclear whether PEEs have a direct effect on children’s educational attainment independent of the family background in general using a nationally representative Chinese dataset.

In addition, there are still some shortcomings in previous research in terms of the form of data and estimation methods. First, most studies have used questionnaire-based cross-sectional data that investigated PEEs and children’s academic achievement at the same time, which failed to exclude the reverse causality between PEEs and children’s current academic performance. The correlation between children’s current academic performance and their eventual educational attainment can make the estimation of the effect of PEEs meaningless. Second, in terms of estimation methods, existing studies do not address the issue of endogeneity. For example, unobservable omitted variables, such as the psychology of comparison from parents and measurement error due to differences in parental expression or performance, can lead to attenuation bias.

Studies showed that PEEs were less dependent on family socio-economic status in Asia than in the West, and Asians tended to hold high educational expectations for their children regardless of their family socio-economic status (Li and Chen, 2007; Liu and Xie, 2016; Li and Xie, 2020). Based on literature and observations, the present study hypothesized that in addition to the indirect impact, the PEEs also have a direct influence on children’s higher education attainment independent of family background in China. Moreover, the endogenous problem may affect the estimation results of the magnitude of the PEE effect. If this is the case, regressing with instrumental variables could result in a significant difference in estimation compared to regressing with PEEs directly. To test this hypothesis, the present study used longitudinal data from CFPS and average CEOI when the children were 10–12 years old as an instrumental variable for PEEs to test the independence effect of PEEs and its magnitude. Furthermore, we sought to explore the PEE effect on different genders and families of various income levels, in order to make the conclusions more comprehensive.

## Literature Review

### The Role of Parental Educational Expectations

The Blau-Duncan Status Attainment Model, which treats education as an intermediate factor in the intergenerational transmission of family dominance, has been recognized by many scholars and has led to discussions about how strengths and weaknesses of family background factors can determine differences in the educational attainment of offspring, and thus ultimately influence the occupational and economic status of children (Blau and Duncan, 1967; Ganzeboom et al., 2003). Among these theses, the Wisconsin School incorporated educational expectations into the Status Attainment Model and showed that they had a significant impact on educational attainment, which was subsequently refined as the Wisconsin Psychosocial Model of Status Attainment (hereinafter referred to as the “Wisconsin model”) (Sewell et al., 1970). PEEs, as part of educational expectations, crucially impact children’s educational attainment (McCoy et al., 2016; Cross et al., 2019; Li and Hu, 2021). Wang et al. (2018b) further argued that PEEs positively predicted Students’ test scores.

Regarding the role of PEEs in the educational attainment of children, there are two main perspectives. The first perspective is that PEEs are an intermediate variable of family background. According to the Wisconsin model, family background is an important factor influencing PEEs. For example, higher PEEs are linked to more investment in education, which raises children’s own educational expectations through their parents’ words and deeds, which in turn leads to better educational attainment (Passeron and Bourdieu, 2002; Qiu, 2012; Liu et al., 2014). The intermediate influence of PEEs has been explored by many Chinese scholars. Yang and Wan (2015), by investigating the education separation of Chinese junior middle school graduates, found that family economic capital was not only positively correlated with PEEs, but that it also indirectly affected children’s academic performance through PEEs. Wang and Shi (2014) showed that family background factors influence children’s individual educational expectations through PEEs, and that eventually the two kinds of educational expectations would affect children’s higher education jointly. Long and Pang (2016) argued that family wealth, home educational resources, and parental education have significant indirect effects on children’s academic achievements.

The second perspective is that PEEs have an impact on children’s educational attainment independent of family background. Sewell and Shah (1967) constructed a model of family background factors, educational expectations, intelligence factors and higher education attainment and concluded that educational expectations have an impact on educational attainment independent of family background factors and intelligence. Based on a sample of junior students in China, Fang and Huang (2019) found that PEEs helped to improve children’s academic performance which in turn affected the possibility of achieving higher educations. By tracing and investigating two individuals with similar family backgrounds in China, Wang and Qi (2014) examined the differences in children’s academic performance under the circumstance that their parents had the same educational level, the family social status and economic conditions, but the PEEs for their children were quite different. The results suggested that higher PEEs significantly improve children’s academic performance, although the sample was too small to generalize to a larger population. Our study, instead, focuses on the independent influence of PEEs on children’s tertiary education attainment across a wide range of families.

Education has always been an important driver of upward mobility for the lower and middle classes (Blau and Duncan, 1967; Wu et al., 2017; Jin et al., 2019). If PEEs have an impact on children’s educational attainment independent of family background, it means that in addition to changing their existing socio-economic status, people can also promote social class mobility by changing their ideas. Influenced by traditional Confucian culture and the long history of imperial examinations, average families in China attach much more importance to education than families in western countries, and this phenomenon is largely independent of family background (Liu and Xie, 2016). Li and Chen (2007) showed that 87.14% of China’s parents who were from poor families expected their children to have a college degree or above, while also hoping that their children could change their living environment by receiving a good education. This notion is underscored by traditional Chinese sayings, such as “a noble son coming from a poor family” and “knowledge changes destiny.” This unique cultural tradition makes it feasible to change ideas to promote social mobility in China and certainly makes sense in an era of widening disparities between the rich and the poor.

### The Inequality Phenomenon of Educational Attainment

As stated above, PEEs may play a great important role in narrowing the gap between different groups. At the macro level, since market-oriented reforms were enacted in China, education has become an important way for people to achieve higher socio-economic status, while the main function of education has changed from eliminating class differences to producing talent for economic growth (Li, 2003). Gender differences in educational attainment have also become one of the main sources of the wage differential between men and women in the labor market which has received considerable scholarly attention (Zhang, 2013). Since the founding of China, gender inequality regarding access to education in China has been on a downward trend (Wu, 2012). One noticeable fact, however, is that the recent educational attainment of Chinese men has been significantly lower than that of women (Wu, 2012). This inequality of educational attainment is worthy of attention not only for females, but also for males.

In addition, the education attainment inequality has also occurred in families of various income levels. Education inequality was an important reason for the widening income gap (Wen, 2007). According to human capital theory, individuals with higher human capital levels have higher labor productivity, so the difference in human capital level leads to an income gap between families (Becker, 1975). Yang and Zhao (2013) found that education expansion helps to control the income distribution gap. Yang and Huang (2010) also found that educational support for low-income groups helped reduce income disparities.

When the Chinese government issued the ‘‘Opinions on Further Reducing the Burden of Homework and Off-Campus Training on Students in Compulsory Education’’ policy (hereinafter referred to as the ‘‘Double Reduction’’ policy) recently, the extracurricular training industry was banned.^1^ As a result, access to extracurricular education for children from low- and middle-income families was curtailed. Therefore, there is an urgent need for a new, cheaper way for children from disadvantaged families to get higher education in China. Thus, the findings of this paper may provide a feasible solution in the current context.

The unequal access to educational opportunities is a reflection of the unequal distribution of resources among different social strata and groups. Therefore, investigating the influencing factors of educational inequality can deepen the understanding of the stratification structure and mobility pattern of Chinese society. With the “Double Reduction” policy as a backdrop, this paper has important practical significance for alleviating social contradictions and maintaining social stability.

## Identifications of Instrumental Variables

### Potential Endogenous Problems

Endogeneity is a common problem in micro-quantitative analysis, and its causes include reverse causality, omitted variables and measurement error. When there is endogeneity, the estimation results can deviate (over or under) from the true parameter values. When we combed through existing studies, we found that endogenous problems have not received much attention.

First, there is a potential reverse causal relationship between PEEs and children’s educational attainment. Most domestic and international studies have used cross-sectional data from single item questionnaires as samples for analysis, such as Thurston et al. (2011), Liu et al. (2014), and Wang and Shi (2014). Pinquart and Ebeling (2020) used meta-analysis and found a positive correlation between PEEs and children’s current academic performance. This implied that children’s current performance can also influence PEEs. If such a correlation is not excluded, the regression of PEEs on children’s final educational attainment would equally explore the influence of children’s current academic performance on the final educational attainment, not PEEs.

Second, there exists other possible omitted variables. For example, comparing mentality may lead parents to have high educational expectations of their children, and result in a downward bias in the estimates (Pinquart and Ebeling, 2020). Similarly, the existence of children’s rebellious psychology can also underestimate the influence of PEEs. These omitted variables may lead to an inconsistent result and the extent of the deviation cannot be predicted.

Finally, neglecting the measurement error can affect the result. In reality, the mechanisms by which PEEs influence children’s higher education attainment are complex, and it only represents a part of PEEs with any single-dimensional question, which might lead to the typical problem of measurement error. In addition to this, differences in parental expressiveness or execution can exacerbate the problem. These measurement errors can lead to a weakening bias in the results, which may underestimate the influence of PEEs on children’s educational attainment.

### College Enrolment Opportunity Index

Instrumental variables are one of the most effective ways to address endogeneity. In this paper, the average CEOI when the children were 10–12 years old was selected as an instrumental variable for PEEs to explore its independent impact on children’s higher education attainment. College enrolment opportunity was an exogenous variable determined by macro factors such as college entrance rates and was not correlated with control variables such as family background and personal characteristics. However, the probability of entering university can influence the parental expectations of their children’s education, and college enrolment opportunities in the past when the children were 10–12 years old would not directly affect the probability of higher education attainment since the children would take the Gaokao at around 18 years old, which meets the exogenous requirements of instrumental variables. We cover these two areas in detail below.

#### College Enrolment Opportunity

Passing the Gaokao is the most important way for residents of China to enter formal higher education. However, the admission score of the Gaokao is determined by the distribution of colleges and universities, population, economy, and allocation of educational resources under the principle of regional equity (Li, 2010), and the examination papers and scoring methods used by different provinces are different resulting in the inability to make cross-sectional comparisons between the admission scores in different provinces. For example, Hebei and Shanxi provinces use the same set of Gaokao papers; however, a Shanxi candidate with a score of 150 in Science in 2020 would have been able to enter a specialized school, while a candidate with the same score in Henan would not have been admitted into higher education.^2^ The difficulty of the Gaokao in different provinces fluctuates and cannot be measured by the admission score. Therefore, we constructed the CEOI to measure this volatility.

Based on the entrance opportunity index with the high school graduates as a benchmark constructed by Li (2010), this paper constructs the CEOI of each province in China since the restoration of the Gaokao and uses it as an instrumental variable to estimate the influence of PEEs on their children’s higher education. First, using high school graduates and college enrolment data for each province from 1977 to 2018 in the China Education Statistics Yearbook, we calculated an entrance opportunity index based on high school graduates for province *i* in year *y*^3^:


(1) $$\hat{i\,n\,d\,e\,x_{i}^{y}}=\frac{e\,n\,r\,o\,l\,m\,e\,n\,t_{i}^{y}}{e\,n\,r\,o\,l\,m\,e\,n\,t_{t\,o\,t\,a\,l}^{y}}÷\frac{g\,r\,a\,d\,u\,a\,t\,e\,s_{i}^{y}}{g\,r\,a\,d\,u\,a\,t\,e\,s_{t\,o\,t\,a\,l}^{y}}$$

where *enrolment* indicates the number of high school enrolments, and *graduates* indicates the corresponding number of high school graduates. If the index is greater than one, it means that the examinees in province *i* have a higher probability (or lower difficulty) of accessing higher education than the national average level in year *y*, and vice versa.

To make the index longitudinally comparable and eliminate the heterogeneity of provinces, we then multiplied the index calculated by Formula (1) by the national enrolment rate of the Gaokao in the corresponding year (see Formula 2), and then subtracted the mean value of the index derived by Formula (2) of each province over the years to construct the CEOI used in this paper^4^ :


(2) $$\tilde{i\,n\,d\,e\,x_{i}^{y}}=\hat{i\,n\,d\,e\,x_{i}^{y}}\times \frac{e\,n\,r\,o\,l\,m\,e\,n\,t_{t\,o\,t\,a\,l}^{y}}{r\,e\,g\,i\,s\,t\,r\,a\,t\,i\,o\,n_{t\,o\,t\,a\,l}^{y}}$$

(3) $$i\,n\,d\,e\,x_{i}^{y}=\tilde{i\,n\,d\,e\,x_{i}^{y}}-m\,e\,a\,n\,\left(\tilde{i\,n\,d\,e\,x_{i}^{1977-2018}}\right)$$

where *registration* indicates the number of people enrolled in the college entrance examination. To control for provincial heterogeneity, we subtracted the CEOI from its mean from the resumption of the Gaokao (year 1977) to year 2018 to ensure the accuracy of the data (see Formula 3).

#### Effectiveness Discussion

Effective instrumental variables require both correlation and exogenous conditions. In our study, the correlation condition refers to the fact that the probability of university acceptance can influence PEEs. Research from the Wisconsin School has suggested that educational expectations are influenced by national policies, educational settings, and labor markets at the macro-level (Andres et al., 2007). From the above calculation process, it is clear that the value of CEOI depends on macro policies such as the college entrance examination enrolment plan, and the instrumental variable satisfies the correlation condition.^5^

Exogenous conditions mean that there is no correlation between the CEOI and other influencing factors, and CEOI would affect the explained variables only through the corresponding endogenous variables (i.e., PEEs). From the first aspect, the probability of entering a university is determined by macro policies, which means it has good externality and would not correlate with other micro factors, such as family backgrounds and individual characteristics. Regarding the second aspect, the past CEOI does not directly affect the college entrance rate of the children when they take the Gaokao at around 18 years old. Because of the sequential nature of education decisions, we assume that the PEEs of children who enter key middle schools would be higher than those of non-key middle schools. In order to avoid this reverse causality, we selected the average CEOI when the children were 10–12 years old (12 years is the node at the beginning of middle school) as the instrumental variable of PEEs to avoid the endogenous problems in the estimation process.

In addition, OLS regression was used to test the exogeneity of the instrumental variable (Table 1). The explained variables of the two models in Table 1 are the children’s education level. The second column contains only the variable of the average CEOI when the children were 10–12 years old, and the third column adds the PEEs variable. It can be seen from the regression results that before controlling for PEEs, the average CEOI of children aged 10–12 was significantly positively correlated with their children’s educational level. However, after controlling for PEEs, the coefficient is no longer significant, and the coefficient of the PEEs variable is significantly positive, indicating that the instrumental variable influences children’s education level only through the path of PEEs. In sum, the instrumental variable has good effectiveness.

**TABLE 1:** OLS model of instrumental variables.

Variables	Education level of children	Education level of children
PEEs		0.062***
		(0.006)
Index	0.436*	0.306
	(0.256)	(0.248)
No. of samples	1,835	1,835

1. Standard errors are in parentheses; 2. *, **, ***indicate significant at 0.1, 0.05, and 0.01 levels, respectively; 3. Index means the average College Enrolment Opportunity Index of children aged 10–12.

## Data and Methodology

In response to the previous endogenous problems, this paper focuses on children’s tertiary education attainment, and re-estimates the problem in terms of both data and estimation methods. We measured the variable of PEEs using the data from the CFPS Children’s Questionnaire (when the children were 10–15 years old); however, the variable of whether the children had obtained higher education was measured by the question answered by samples in adulthood. In terms of estimation methods, the average CEOI when the children were 10–12 years old was selected as an instrumental variable for PEEs to estimate the extent to which PEEs affected the children’s higher education attainment after controlling for other factors, such as family background.

### Data Description

Apart from the instrumental variables, all data used in this paper were taken from CFPS 2010 to 2018. The CFPS is a nationwide survey project hosted by the China Social Science Research Centre of Peking University, with data from 25 provinces, municipalities and autonomous regions in China at three levels: individual, household, and community, with wide coverage and more representative sampling data. Data collection began in 2010, from which all sample members and their offspring were permanently tracked. The CFPS tracking survey enables us to obtain the PEEs when their children were minors and the educational level when the children became adults, which is the core explanatory variable and explained variable in this paper, respectively. As such, we circumvented part of the reverse causality problems from the perspective of data.

The explained variable in this paper was the children’s higher education attainment, with a value of 1 for attaining and 0 for unattaining.^6^ As suggested by China’s Compulsory Education Law, children enter primary school at the age of 6, followed by 9 years of compulsory primary education and 3 years of secondary education; they then take the Gaokao (the college entrance examination in China) at the age of 18 if there are no interruptions (Wu, 2010). Meanwhile, we estimated that the average age at which rural and urban residents take the Gaokao is 18.08 years old by using 61,115 samples from the 2013 Chinese Household Income Project. Due to the expansion of college enrolment in China, the enrolment rate of the Gaokao has exceeded 90%.^7^ In order to avoid any bias in the measurement of the sample’s higher education attainment due to many samples being in the third year of high school, the sample of 18-year-olds was excluded, and we selected a total of 1,835 samples from the survey conducted from 2010 to 2014 who were 10--15 years old and had clear PEEs.^8^

The core explanatory variable in this paper is PEEs, which are derived from the CFPS Children’s Questionnaires, where parents were asked to answer the question ‘‘What is the highest level of schooling you would like your child to complete?’’ Eight options were offered ranging from illiterate to PhD.^9^ The ninth option was ‘‘no need to study’’ which was treated the same as ‘‘illiterate’’ in this research. As the baseline households participating in the CFPS between 2010 and 2014 would sometimes change their answers to this question, we used the mean value of PEEs between 2010 and 2014 as a measure of PEEs and transformed PEEs (level of education) into the corresponding expected number of years of schooling to avoid the non-continuous integer variable.^10^

To ensure the accuracy of the data, we checked the answers which were “unsuitable” or “I don’t know,” finding that most of the parents of these samples were illiterate or primary school educated, meaning that no highly educated parents refused to answer the question, which would have resulted in a large number of abnormal phenomena in the sample.

The control variables were the family background factors. Most of the existing literature has used occupational status and parents’ education level to indicate family background (Blau and Duncan, 1967; Liu et al., 2014; Wang and Shi, 2014). In our study, we used the higher value of the International Socioeconomic Index (ISEI) of the samples’ father or mother to indicate the occupational status of the parents. The values of ISEI usually ranged from 20 to 80, with higher values indicating higher socio-economic status in that occupation. We used the higher education level of either the father or mother to indicate the level of education of the parents and converted it to years of schooling in the same way as above. In addition, we controlled for a range of individual characteristics such as gender, year of birth and type of hukou.^11^ The descriptive statistics of the variables used in this paper are shown in Table 2.

**TABLE 2:** Descriptive statistics.

Variables	Values	No. of samples	Means	*SD*	Min	Max
Children’s higher education attainment		1,835	0.374	0.484	0	1
Parental educational expectations (PEEs)		1,835	15.94	3.391	0	23
Parents’ ISEI		1,835	33.16	13.983	20	88
Parents’ schooling years		1,835	7.21	4.316	0	23
Year of birth		1,835	1997.53	1.156	1996	1999
Gender	Female	1,835	0.49	0.5	0	1
	Male	1,835	0.51	0.5	0	1
Type of hukou	Agricultural hukou	1,835	0.83	0.378	0	1
	Non-agricultural hukou	1,835	0.17	0.378	0	1

### Methodology Specification

This paper focuses on the independent influence and degree of the PEE effect in childhood on children’s higher educational attainment in adulthood. We first use the Logit model to explore whether PEEs have an independent influence on their children’s higher education attainment after controlling for family background and personal characteristics variables:


(4) Yi*=β0+β1⁢e⁢x⁢p⁢e⁢c⁢t⁢a⁢t⁢i⁢o⁢ni+β0⁢Xi+μi

(5) Yi=1(Yi*>0)

where *Y_i_* is individual*i*′s educational level, and *expectation*_*i*_ is the PEE. *X_i_* includes a series of family background variables, such as parents’ years of schooling and parents’ ISEI, and children’s personal characteristic variables, such as age, gender, and household type.

Later, in order to address the endogeneity problem and investigate the degree of influence, we used the instrumental variable to measure PEEs. Therefore, the variable of PEEs in Formula (4) is estimated as follow:


(6) $$e\,x\,p\,e\,c\,t\,a\,t\,i\,o\,n_{i}=\gamma_{0}+\gamma_{1}\,i\,n\,d\,e\,x_{i}+\gamma_{0}\,X_{i}+\varepsilon_{i}$$

where *index*_*i*_ is the average value of the CEOI when the children were 10–12 years old. This method is the so-called IV-Logit model.

## Regression Results and Analysis

### Main Regression Results

As the explained variables are discrete (taking 0 or 1), we used the Logit model for regression to improve the estimation efficiency. The main estimation results are reported in Table 3, where models (1) and (2) are estimated without instrumental variables, and models (3) and (4) are estimated with instrumental variables. We first compared models (1) and (2). The difference in the setting of the two models is that model (2) includes family background factors—the parents’ ISEI and the parents’ schooling years—as explanatory variables. If PEEs are only an intermediary variable between family background factors and children’s higher educational attainment, then the coefficient on PEEs will change from significant to insignificant when the model includes family background factors as explanatory variables. Conversely, if PEEs have an independent role in determining children’s higher education attainment, the inclusion of family background factors would not affect the significance of the coefficient on PEEs. In particular, the smaller the change in the coefficient of PEEs when the family background factor is included, the more independent the impact of PEEs would be.

**TABLE 3:** (a) Logit and IV-Logit models of children’s higher education attainment.

	(1) Logit	(2) Logit	(3) IV-Logit (2nd stage)	(4) IV-Logit (2nd stage)
Explained variable	Children’s higher education attainment			
PEEs	0.120***	0.105***	0.321**	0.285*
	(0.017)	(0.017)	(0.024)	(0.167)
Year of birth	0.322***	0.343***	0.288***	0.311***
	(0.047)	(0.048)	(0.049)	(0.053)
**Gender**				
Male	−0.539***	−0.538***	−0.471***	−0.487***
	(0.105)	(0.106)	(0.109)	(0.111)
**Type of hukou**				
Agricultural	1.001***	0.700***	0.698***	0.555***
Hukou	(0.132)	(0.144)	(0.230)	(0.187)
Parents’ ISEI		0.670***		0.429*
		(0.159)		(0.255)
Parents’		0.603***		0.397*
Schooling years		(0.156)		(0.228)
No. of samples	1,835	1,835	1,835	1,835

**(b) First stage of IV-Logit models**				
	**(1) Logit**	**(2) Logit**	**(3) IV-Logit**	**(4) IV-Logit**

Explained variable			PEEs	PEEs
Average CEOI	−	−	6.402***	5.491***
	−	−	(1.361)	(1.348)
Individual characteristics	−	−	Y	Y
Family backgrounds	−	−	N	Y
*F*-values	−	−	19.00	22.04
No. of samples	−	−	1,835	1,835

1. Standard errors are in parentheses; 2. *, **, ***indicate significant at 0.1, 0.05, and 0.01 levels, respectively; 3. Average CEOI means the average College Enrolment Opportunity Index of children aged 10–12; 4. Variables of personal characteristics included: gender, year of birth, and type of hukou; variables of family backgrounds included: parents’ ISEI and parents’ schooling.

Comparing the results of models (1) and (2) in Table 3, the coefficient on PEEs remains significant at the 1% level after the inclusion of the parents’ ISEI and the parents’ schooling years, and the coefficient does not change much (from 0.120 to 0.105).^12^ This suggests that although there is some influence of family background factors on PEEs, PEEs still have a role in children’s educational attainment independent of family background factors, and this independency should not be ignored.

We then compared models (1) and (3), where the difference between the two models is whether instrumental variables are used for regression. If there were serious endogenous problems with a direct regression on PEEs, the coefficients on PEEs estimated by the two models would differ significantly. By comparing the results of models (1) and (3) in Table 3, we found that the coefficient of PEEs increases from 0.120 to 0.321 after regression with instrumental variables and is still significant at the 5% level. This result confirms our previous speculation that there is a serious endogenous problem in regressing PEEs directly. After addressing the endogeneity issue, we found that the effect of PEEs on children’s educational attainment is much larger than previously estimated. At this point, the marginal effect at the sample mean is 0.07 (*p* < 0.05), indicating that for every year of increase in PEEs, the probability of their children attending university increases by 7 percentage points.

Finally, we compared models (3) and (4). Similarly, the difference between models (3) and (4) in terms of setting was the inclusion of family background factors. Comparing the results of models (3) and (4) in Table 3, we found that although the coefficient on PEEs decreased from 0.321 to 0.285,^13^ the latter is still significant at the 10% level. After addressing the endogeneity issue, PEEs remained unaffected by family background, suggesting that the independent impact of PEEs is robust through IV-Logit estimation, and it also implies that the results of IV-Logit estimation are robust.

Within all four regressions, the year of birth was used as a control variable in all models to control the heterogeneity of individuals born in different years. The coefficient of the male variable was significantly negative, indicating that the tertiary education attainment of males is currently lower than that of females. The education level of urban residents was generally higher than that of rural residents. In models (2) and (4), where the family background was included, both parental economic status and education level significantly and positively affected the probability of children’s tertiary education attainment.

### For Gender Differences

Our study sought to explore the impact of parental educational expectations on their children’s higher education attainment on different genders to highlight a new path for the process of equalization of education during rapid economic development. Thus, we added an interaction term of PEEs and children’s gender in model (4) (Table 3), which is the variable where PEEs interact with the male dummy variable. If the coefficient of the interaction term is significant, it means that the effect of PEEs is gender-differentiated; conversely, if the coefficient of the interaction term is not significant, the effect of PEEs is not gender-differentiated.

Table 4 presents IV-Logit regressions of the differences in the impact of PEEs on higher education attainment for children of different genders. In the second stage of the regression, while the coefficient of the PEEs variable remained significantly positive (row 3, column 2), the gender variable and the interaction term of PEEs and males were not significant, indicating that PEEs still positively affected children’s tertiary education attainment and that there was no significant gender difference in the effect on children’s tertiary education attainment. Notably, the coefficient for males in the model (4) of Table 3 was significantly negative, but after the interaction term was added, the coefficient was not significant, so we further investigated whether the lower probability of higher education attainment for males than females was due to the different educational expectations of parents for their children by gender. The first stage regression in Table 4 shows that PEEs did not differ significantly according to the gender of their children (row 2, column 4). Therefore, the change of the coefficient of gender variable may have been caused by other reasons. We did not conduct an in-depth investigation, and this phenomenon does not affect the conclusion that there was no gender difference regarding the influence of PEEs on their children’s higher education.

**TABLE 4:** (a) Second stage of IV-Logit models of gender differences.

Explained variables	PEEs	Average CEOI	Gender (Male)	PEEs × Male	Other control variables	No. of samples
Children’s higher	0.412**	−	2.758	−0.201	Y	1,835
Education attainment	(0.189)	−	(2.111)	(0.131)		

(b) First stage of IV-Logit models						
PEEs	−	5.491***	−0.217	−	Y	1,835
	−	(1.348)	(0.153)	−		

1. Standard errors are in parentheses; 2. *, **, ***indicate significant at 0.1, 0.05, and 0.01 levels, respectively; 3. Average CEOI means the average College Enrolment Opportunity Index of children aged 10–12; 4. Other control variables included: gender, year of birth, type of hukou, parents’ ISEI, and parents’ schooling.

### For Families of Varied Income

We divided the sample into low-, middle-, and high-income households by referring to the parental ISEI at two nodes in the 25th and 75th quartiles and divided the sample into six groups by combining the sample’s type of hukou. The IV-Logit regression and the sample means in each group were used to test the degree of influence of PEEs on different households (Table 5). Table 5 shows that the PEE effect is greater when the initial probability of gaining tertiary education approaches 50%. This means that the effect of increased PEEs is more significant for low- and middle-income households which are at the margins (i.e., the initial probability is 50%) of gaining tertiary education. For example, for middle-income households in rural areas, the initial probability of the children obtaining higher education is 46.69%, and it increases by 7.11 percentage points if their parents increase PEEs by 1 year. In contrast, the effect is 4.58 percentage points when the initial probability is 77.50%.

**TABLE 5:** IV-Logit models of families of varied income.

Parents’ ISEI	Probability of obtaining higher education of children with agricultural hukou			Probability of obtaining higher education of children with non-agricultural hukou		
	Initial probability	1 year increasing of PEEs	Differences	Initial probability	1 year increasing of PEEs	Differences
Low	34.75%	41.45%	6.70%	59.80%	66.42%	6.62%
Middle	46.69%	53.80%	7.11%	69.68%	75.34%	5.66%
High	62.34%	68.76%	6.42%	77.50%	82.08%	4.58%

*1. Low-, meddle-, and high—income households are defined as those with parents’ ISEI below the 25th percentile, between the 25 and 75th percentile, and above the 75th percentile of the samples, respectively.*

With extracurricular education for low- and middle-income families now banned by the “Double Reduction” policy, the conclusion of this paper provides strong theoretical support for such families who hope their children can attain higher degrees without spending too much money. PEEs are not necessarily a proxy for the economic status of the household; however, the positive effect of PEEs on their children’s higher education attainment can also be shown by the model (4) in Table 3, which reveals another possible contributor to the emergence of “a noble son coming from a poor family,” i.e., parental expectations.

## Discussion

As an important investment in human capital accumulation, the importance of education has been recognized by most, if not all societies. As for the influential factors of educational attainment, there is no lack of discussion asserting that family background or parents’ economic status positively influences PEEs, which in turn positively influence children’s education attainment (Ganzeboom et al., 2003; Wang and Shi, 2014; Pinquart and Ebeling, 2020). However, there are still cases in which parents with low economic status want their children to improve their living environment by receiving a good education (Li and Chen, 2007), which in turn motivates their children to study hard and obtain a higher level of education. In addition, many of the studies on parental expectations and children’s educational attainment have used data with unavoidable endogenous problems, such as reverse causality, omitted variables, and measurement errors (Thurston et al., 2011; Liu et al., 2014; Wang and Shi, 2014). If these endogeneity issues are ignored, the estimates of the effect of PEEs on children’s educational attainment may be biased.

This study used CFPS tracking survey data from 2010 to 2018 to estimate whether PEEs have a direct influence on children’s higher education attainment independent of family background factors besides the intermediate effects. To address the endogenous problems, we explored the magnitude of that effect using an average CEOI of children at age 10–12 as an instrumental variable. This study, however, is the first one we know of that uses the instrumental variable method to explore the PEE effect in the Chinese context. In contrast to existing research, we found that PEEs have a significant positive effect on children’s higher education attainment after controlling for family background factors such as parents’ occupational status and parents’ schooling years, which means that in addition to the indirect effect, PEEs also have a direct positive effect on children’s higher education attainment independent of family background.

What’s more, there is a strong positive correlation between the average CEOI when the children were 10–12 years old and PEEs. After using this instrumental variable, the PEEs have a far greater impact on the children’s higher education attainment than Logit estimates. The reasons may be due to the influence of Confucianism and the imperial examination system; Chinese tend to attach more importance to education and the cultural atmosphere than people in Western countries (Kipnis, 2011). Therefore, the link between PEEs and family background may not be as strong as in Western countries. Other conclusions drawn from the coefficients of control variables regarding the higher educational attainment of female/urban residents surpassing male/rural residents, and parents’ ISEI or years of schooling being positively related to the children’s tertiary education attainment, were consistent with previous research (Charles, 2011; Wu, 2012; Long and Pang, 2016), increasing the credibility of the present study.

Further, we examined the PEE effect on different genders. First, the first-stage regression for the male and female samples showed that there were no gender differences in PEEs. By investigating the parental educational expectations of primary and secondary school students in Urumqi and Changchun, Yang Chunhua (2006) found that both boys and girls had the same educational expectations as their parents, which was verified by our regressions. Second, the results showed that there were no significant differences in the effect of PEEs on the probability of higher education attainment for children of different genders from the second-stage regression. Although education inequality between genders has declined due to the educational expansion around the whole country, poverty still contributed to gender inequality in rural areas (Yang et al., 2014). A cheap and effective way to eliminate education inequality between genders is in urgent need. Our findings provide theoretical support for the PEE effect in solving this problem.

Finally, we explored the PEE effect on families of varied incomes. The results showed that the effect of increased PEEs was particularly significant for low- and middle-income households, especially for middle-income households in rural areas, where each 1-year increase in PEEs increased the probability of children obtaining tertiary education by 7.11 percentage points. Given the new “Double Reduction” policy in China, the educational incentives of youth from low-income families may be weakened, which may lead to a further rise in class consolidation (Lv and Wu, 2013). The findings of this paper provide an important theoretical basis for encouraging parents in low- and middle-income families to have high educational expectations for their children and express them appropriately to their children. Parents who value their family culture and raise their children’s education expectations can enhance their children’s motivation to learn and improve their learning efficiency, which can lead to higher educational achievement.

The results of our analysis do not imply that PEEs are completely independent of family background factors and are determined by the difficulty of access to university. We stress that PEEs are influenced by many other factors besides family background, of which the college entrance opportunity is only one of these factors. However, as college entrance opportunity is exogenous, it can be seen as a useful tool for isolating the impact of PEEs on children’s higher education attainment. The increase of parents’ educational expectations brings higher benefits to their children’s higher education, and raising parent awareness of the importance of education may be a new way to increase future human capital accumulation.

While this paper focuses on the independent influence of PEEs on children’s higher education attainment, it still neglects important questions such as how PEEs influence children’s educational attainment at other stages of education; or how inconsistencies in the “expression” or “action” of PEEs for their children may lead to differences in the educational achievement of children with the same parental expectations. These questions offer directions for future research.

## Data Availability Statement

The original contributions presented in the study are included in the article/supplementary material, further inquiries can be directed to the corresponding author/s.

## Author Contributions

TL was the primary lead author of the article and did most of the data cleaning, regressions, and much of the analyses. After finished the first draft, FL helped to write up the manuscript and contributed much to the revision and submission of the manuscript. YH modified the text part and made a lot of contributions to the empirical result analyses. All authors contributed to the article and approved the submitted version.

## Conflict of Interest

The authors declare that the research was conducted in the absence of any commercial or financial relationships that could be construed as a potential conflict of interest.

## Publisher’s Note

All claims expressed in this article are solely those of the authors and do not necessarily represent those of their affiliated organizations, or those of the publisher, the editors and the reviewers. Any product that may be evaluated in this article, or claim that may be made by its manufacturer, is not guaranteed or endorsed by the publisher.

## Footnotes

1 See the website of the Ministry of Education of the People’s republic of China: http://www.moe.gov.cn/jyb_xxgk/moe_1777/moe_1778/202107/t20210724_546576.html.

2 In 2020, the Gaokao admission score of Science in Shanxi Province was 130, and that of Henan Province was 180. The examinee with 150 points in Science in Shanxi Province is more likely to enter college, while the same score in Henan province would have been excluded from higher education. Data source: Education Department of Shanxi and Henan Province.

3 Li (2007) suggests the feasibility of using the number of high school graduates to replace the number of applicants in Gaokao because it is difficult to obtain the data of the number of applicants in each province.

4 Data source: compiled from China education public data.

5 Moreover, the methodological proof can be seen in the first-stage regressions of model (3) and (4) in Table 3. The coefficient of Index is significant positive, which indicates that the average CEOI when the children were 10–12 years old is positively related to PEEs.

6 Subject to the year of the CFPS tracking survey, in 2018, the oldest child of the sample aged older than19 and who had answered the Children’s Questionnaire was 23 years old.

7 Source: Compiled from public data on education in China. The national college enrolment rate for the 2020 is 90.57%.

8 To ensure the sample is 19 years old or older by 2018, samples aged 11–15 were retained in CFPS 2010; In CFPS 2011, samples aged 12–15 were retained; In CFPS 2012, samples aged 13–15 were retained; In CFPS 2014, only samples aged 15-years-old were retained.

9 The answers to this question in the CFPS questionnaire were assigned the following values: 1. illiterate/semi-literate; 2. primary school; 3. junior high school; 4. high school; 5. college; 6. undergraduate; 7. master’s degree; 8. doctorate; 9. no need to study.

10 Years of education were converted as follows: 0 year for illiteracy; 6 years for primary school; 9 years for junior school; 12 years for high school; 15 years for specialized school; 16 years for university; 19 years for master’s degree; and 23 years for doctorate. The conversion method for parents’ education level is the same.

11 Hukou is the unique household registration system in China and has two types: agricultural hukou and non-agricultural hukou.

12 The two coefficients were significantly different according to the Seemingly Unrelated Model (SUEST) test, which was not reported in detail due to space constraints, and the same below.

13 SUEST test showed that there was no significant difference in these two coefficients.
